# Prognostic Models for Renal Cell Carcinoma in the Era of Immune Checkpoint Therapy

**DOI:** 10.7759/cureus.30821

**Published:** 2022-10-29

**Authors:** Andreea Parosanu, Ioana Miruna Stanciu, Cristina Pirlog, Cristina Orlov Slavu, Horia Cotan, Cristian Iaciu, Ana Maria Popa, Mihaela Olaru, Oana Moldoveanu, Baston Catalin, Cornelia Nitipir

**Affiliations:** 1 Oncology, Elias Emergency University Hospital, Bucharest, ROU; 2 Medical Oncology, Elias Emergency University Hospital, Bucharest, ROU; 3 Urology, Fundeni Clinical Institute, Bucharest, ROU

**Keywords:** predictive risk factors, serum biomarkers, immune check-point inhibitor, risk factor model, renal cell carcinoma (rcc)

## Abstract

With recent advances in oncology, immune checkpoint inhibitors (ICIs) have become a milestone in immuno-oncology. Unfortunately, although ICIs have demonstrated improved clinical efficacy in a broad spectrum of cancers, many patients do not respond to this newer therapy. As a result, it is crucial to identify predictive factors of response to immunotherapy in patients with kidney cancer. This review discusses the research investigating potential biomarkers of response to ICIs in renal cell carcinoma.

## Introduction and background

Renal cell carcinoma (RCC) is the most common type of renal malignancy, contributing 2.2% of the total number of new cancer cases diagnosed in 2020 [1]. Even though clinical staging is essential to help plan initial treatment, many other factors can predict survival and guide treatment options. They represent valuable tools for a successful outcome in individual treatment. While surgery remains an essential curative treatment option for stages I-III RCC, systemic therapy is the first option for stage IV [2].

The treatment of advanced kidney cancer has evolved dramatically over the past 40 years. In the 1980s, interferon was the only effective therapeutic option for locally advanced or metastatic RCC. The paradigm for treating stage IV RCC has changed over the years with the introduction of tyrosine kinase inhibitors and immune checkpoint inhibitors (ICIs) [3]. With so many therapies available to treat advanced kidney cancer and many more in development, it is essential to find prognostic factors for personalized oncological treatment.

This review aims to answer the following questions: are the standard prognostic models still relevant in the era of immune checkpoint therapy? And which patients will benefit the most from immunotherapy?

## Review

We reviewed the PubMed database from January 2018 to August 2022 to identify relevant studies evaluating prognostic models for patients with renal cell carcinoma treated with immunotherapy. The established inclusion criteria included only English-language research articles that explored the development or validation of prognostic biomarkers of response to modern immunotherapy in advanced and metastatic renal cell carcinoma. From the 97 results found, 38 studies met our criteria. 

Improving risk stratification models

In the early 1990s, Motzer et al. developed the Memorial Sloan-Kettering Cancer Centre (MSKCC) prognostic model for patients with metastatic kidney cancer treated as initial systemic therapy with cytokine interleukin two (IL2) or interferon-alpha (IFN-α). This risk model has been proposed and validated to predict survival based on five pretreatment features: low-performance status, time from diagnosis to treatment interval of less than one year, anemia, elevated lactate dehydrogenase, and corrected serum calcium levels. In addition, patients were categorized according to the number of risk factors into three categories: favorable, intermediate, and poor [4]. Lately, in the era of vascular endothelial growth factor (VEGF) targeted therapy was developed the International Metastatic Renal Cell Carcinoma Database Consortium (IMDC) model. This score was designed by adding two new variables (platelet and neutrophil count) to the MSKCC score [5].

When these two scores were proposed, cytokines and anti-VEGF targeted agents were the standard of care for metastatic renal cell carcinoma. However, since 2015 immune checkpoint inhibitors have changed the treatment paradigm in advanced kidney cancer. So are the previous prognostic models still accurate? Immune checkpoint pivot trials only evaluated the IMDC risk status [6]. Thus, we must reevaluate these models and identify new risk factors that make patients with advanced or metastatic kidney cancer more likely to respond to immunotherapy.

Sagie et al. recently proposed an updated risk stratification model in patients with metastatic kidney cancer treated with checkpoint inhibitors. They demonstrated that in addition to the previous IMDC risk factors, the presence of liver metastasis and the absence of the surgical removal of the primary tumor are negative predictive markers [7]. Several studies have also explored the relationship between sites of metastases and prognosis in RCC patients. Two clinical trials confirmed that liver and central nervous system metastases at diagnosis were solid and independent adverse prognostic factors for survival in RCC patients [8,9]. Moreover, Internò et al. suggested that PD-1 is poorly expressed in brain metastases and immune checkpoint inhibitors seemed less effective in these patients [10].

For early-stage RCC, surgery remains the mainstay of curative therapy. However, advanced RCC needs both local and systemic treatments. Complete cytoreductive surgery in RCC has been associated with better outcomes in patients with good performance status, clear cell subtype histology, and without cerebral metastasis. The goal of surgical treatment was to reduce tumor burden and to achieve negative surgical margins in order to improve response to ICIs and survival [11].

The role of PD-L1 as a predictive or prognostic marker

Up to 30% of RCC overexpressed PD-L1 [12]. Higher expression of PD-L1 was associated with adverse clinicopathological features: poorly differentiated cells, necrosis, and sarcomatoid differentiation [13]. In some malignancies, the tumor PD-L1 expression can predict the response to anti-PD-1/PD-L1 therapy. Thus, in metastatic RCC, the role of PD-L1 as a predictive or prognostic marker is unclear. We reviewed the ICIs in first-line renal cancer trials to evaluate if PD-L1 expression matters (Table 1).

**Table 1:** Pivotal trials of immune checkpoint inhibitor treatments in advanced RCC OSR = overall survival rate, PFS = progression-free survival, ORR = overall response rate, OS = overall survival, PD-L1 = programmed death-ligand 1, ITT population = intention to treat population

Author	Trial	Subgroup	12-month OSR				Median PFS		
Rini et al., 2019 [14]	KEYNOTE-426		Pembrolizumab+ Axitinib		Sunitinib		Pembrolizumab+ Axitinib		Sunitinib
		PD-L +	90.1%		78,4%		15.3 months		8.9 months
		PD-L -	91.5%		78.3%		15.0 months		12.5 months
		Sarcomatoid features	83.4%		79.5%		median not reached		8.4 months
Choueiri et al., 2020 [15]	JAVELIN Renal 101	Subgroup	ORR				Median PFS		
			Avelumab+ Axitinib		Sunitinib		Avelumab + Axitinib		Sunitinib
		PD-L +	55.2%		25.5%		13.8 months		7.2 months
		Overall population	51.7%		25.7%		13.8 months		8.4 months
		Sarcomatoid features	46.8%		21.3%		7 months		4 months
Motzer et al., 2018 [16]	CheckMate 214	Subgroup	ORR				Median PFS		
			Nivolumab+Ipilimumab		Sunitinib		Nivolumab +Ipilimumab		Sunitinib
		PD-L +	69%		24%		22 months		5.9 months
		PD-L -	54%		21%		11 months		10.4 months
		Sarcomatoid features	56.7%		19%		8.4 months		4.9 months
Rini et al., 2019 [17]	IMmotion 151	Subgroup		OS			Median PFS		
				Atezolizumab + Bevacizumab		Sunitinib	Atezolizumab + Bevacizumab	Sunitinib	
		PD-L +		34 months		32.7 months	11.2 months	7.7 months	
		ITT population		33.6 months		34.9 months	11.2 months	8.4 months	
		Sarcomatoid features		21.7 months		15.4 months	8.3 months	5.3 months	
Motzer et al., 2015 [18]	CheckMate 025	Subgroup	OS						
			Nivolumab				Everolimus		
		PD-L +	21.9 months				18.8 months		
		PD-L –	26.8 months				20.3 months		
Choueiri et al., 2021 [19]	CheckMate 9ER	Subgroup	Median PFS						
			Nivolumab+ Cabozantinib				Sunitinib		
		PD-L +	11.9 months				4.7 months		
		PD-L -	17.7 months				9.3 months		
		Sarcomatoid features	10.3 months				4.2 months		

CheckMate 025 was the first study to evaluate the use of PD-L1 expression as a biomarker of response to immunotherapy in advanced kidney cancer. This trial reported that nivolumab improved overall survival (OS) compared to everolimus regardless of PD-L1 expression (21.9 months versus 18.8 months in the PD-L1 positive arm, and 26.8 months versus 20.3 months in the PD-L1 negative arm). However, a positive PD-L1 expression was associated with an unfavorable prognosis because all patients who expressed PD-L1 more than 1% had worse outcomes in both treatment lines [18].

Furthermore, ICIs plus tyrosine kinase inhibitors (TKIs) were tested in three phase III trials, including KEYNOTE-426, JAVELIN Renal 101, and CheckMate 9ER. These studies confirmed increased overall survival rates (OSR) in the ICI+TKI arm irrespective of PD-L1 status [14,15,19].

On the contrary, the CheckMate 214 trial, a phase III study of nivolumab and ipilimumab versus sunitinib, has shown remarkable overall response rates of first-line immunotherapy-based combination in patients expressing PD-L1 over 1% (69% versus 24%) [16].

The combinations of ICIs with angiogenesis inhibitors have demonstrated similar results. For example, in the IMmotion151 trial, atezolizumab combined with bevacizumab (an anti-VEGF monoclonal antibody) showed improved clinical outcomes compared with sunitinib in the PD-L1-positive population. The progression-free survival (PFS) was 11.2 months in the atezolizumab plus bevacizumab versus 7.7 months in the sunitinib arm [17].

Post-hoc analyses of pivotal clinical trials for first-line metastatic kidney cancer included immunotherapy efficacy in tumors with sarcomatoid features. About 5-20% of advanced kidney cancers harbor sarcomatoid differentiation, the most clinically aggressive phenotype. Current studies have shown that RCC with sarcomatoid features may express even higher levels of PD-L1. Therefore, ICIs can be considered a potential therapeutic option [20]. Exploratory analysis of the abovementioned trials confirmed the efficacy of ICIs among patients with sarcomatoid histology. For example, updated findings from the CheckMate 214 trial demonstrated the promising efficacy of nivolumab plus ipilimumab compared to sunitinib (ORR 56.7% versus 19%) in RCC with sarcomatoid features [21]. In addition, in the subgroup of patients with sarcomatoid features from the CheckMate 9ER trial, the combination of nivolumab plus cabozantinib doubled PFS compared to sunitinib (10.3 months versus 4.2 months) [19].

Recent data reported that the prognostic significance of PD-L1 expression is not restricted to clear cell histology [22]. In addition, some studies showed higher levels of PD-L1 in non-clear cell RCC histological subtypes (papillary or chromophobe) than in clear cell RCC [23,24]. It has already been established that non-clear cell RCCs have a more aggressive clinical course. Moreover, in these tumors, PD-L1 positivity was significantly associated with adverse tumor features such as higher TNM stage and Fuhrman grade and reduced overall survival [25].

Other distinctive renal cell carcinoma histological features are inherited forms of kidney cancer. For example, the mutation in the fumarate hydratase gene is associated with hereditary leiomyomatosis and renal cell carcinomas [26]. In addition, despite the currently limited data, PD-L1 positive expression in patients with this hereditary syndrome was associated with higher pathological TNM stages, higher tumor grades, and increased cancer-specific mortality. However, despite the aggressive tumor characteristics, these PD-L1-positive tumors may benefit from anti-PD-1 therapy [27].

So, is PD-L1 expression assessment required for RCC treatment? Currently, the PD-L status is poorly understood and does not play a role in choosing immunotherapy. Further follow-up of these trials is needed to confirm the advantages mentioned above.

Other potential biomarkers

Because kidney cancers are highly immunogenic and vascularized tumors, immunotherapy has shown great potential in using tumor-infiltrating immune cells as promising biomarkers of response to therapy. Several studies have illustrated the robust association between tumor immune microenvironment and the response to immunotherapy. Therefore, many researchers collected data from The Cancer Genome Atlas (TCGA) database and developed a range of immunogenomic landscape signatures to predict immunotherapy response. These immuno-scores, including TMB (tumor mutational burden), PD-L1, CTLA4, or immune-related genes (HLA-B, HLA-A, HLA-DRA), expand knowledge in tumor immune status and provide a potent prediction tool for the future [28,29].

With the approval of ICI blockade in metastatic RCC, is there a biomarker for CTLA-4? There is strong evidence that the DNA methylation of the gene encoding for the CTLA4 protein predicts response to anti-PD-1 and anti-CTLA-4 in patients with melanoma and RCC. CTLA4 methylation status can be quantified using immunohistochemistry and methylation-specific PCR. Klümper et al.'s findings suggest that the CTLA4 hypomethylation status may predict favorable outcomes in RCC patients treated with immunotherapy [30].

There is a strong relationship between inflammation and cancer. Chronic inflammation promotes immune evasion and creates a microenvironment that sustains angiogenesis, tumor initiation, survival, and proliferation. On the other hand, tumor-associated inflammation can lead to aberrant epigenetic alterations that also contribute to the promotion of its growth and survival [31]. RCC is a pro-angiogenic tumor type, as demonstrated by the efficacy of anti-angiogenic agents. RCC is also considered a highly immunogenic tumor. Immunotherapeutic strategies showed efficient activity, including high-dose interleukin-2 or interferon-alpha and, more recently, immune checkpoint inhibitors [32].

There are several inflammation-related plasma biomarkers, such as prognostic nutritional index (PNI), eosinophils levels, lymphocyte-platelet ratio (PLR), and lymphocyte-neutrophil ratio (NLR), which can be affordable and readily available biomarkers of the response to immunotherapy in patients with kidney cancer. NLR and PLR represent a balance between tumor inflammation and immune status. Therefore, an increased NLR is associated with a lower probability of achieving an adequate anti-tumor immune response [33,34]. A meta-analysis including patients with metastatic RCC treated with ICIs suggested that elevated NLR and PLR were associated with unsuccessful treatment outcomes. However, a lower NLR following treatment with ICIs indicated better overall survival [35]. On the other hand, Herrmann et al. demonstrated that elevated blood eosinophil levels within the first six weeks of therapy were associated with improved PFS and OS in patients with RCC treated with nivolumab. This study shows that the blood eosinophils count was not an inflammation response but an allergic response [36].

Another important prognostic factor is the patient's immune-nutritional status. Several studies reported an association between body mass index (BMI), prognostic nutritional index (PNI), and the response to immunotherapy. [37,38]. PNI is calculated using albumin and lymphocyte counts and reflects the relationship between nutritional status and systemic inflammation. There is a vicious cycle between nutrition and inflammation. Hypoalbuminemia may lead to cachexia and impaired immune function [39]. For example, Peng et al. showed that low PNI was an independent poor prognostic factor for response to ICIs in RCC [40].

Moreover, there is an established link between obesity, inflammation, and cancer. Obesity may cause one-quarter of cancer deaths. Obesity also predisposes to inflammation and impacts the response to immunotherapy by activating pro-inflammatory mediators such as interleukin 6 (IL-6) and tumor necrosis factor-alpha (TNF α) [41]. Aurilio et al. have investigated the obesity paradox for cancer immunotherapy. Their findings demonstrated that even if obesity increased the risk of kidney cancer, patients with RCC and high body mass index (BMI over 25 kg/m2) responded better to immunotherapy with TKI+ICI [42]. However, research in this field is just emerging, and further research needs to be done to fully understand how obesity modulates the immune system and influences the efficacy of immunotherapy.

## Conclusions

The prognosis of advanced RCC was primarily based on pathological staging, histological type, grade, and patient performance status. Today, we use risk stratification models to evaluate prognosis and improve treatment. Thus, based on the data available, there are no established predictive biomarkers to distinguish patients most likely to respond to ICIs in RCC. We look forward to more successful research on the obesity paradox, and to further developing potential biomarkers, such as molecular signatures, to maximize the benefit of patients from ICIs.
